# Effective vaccination strategy using SARS-CoV-2 spike cocktail against Omicron and other variants of concern

**DOI:** 10.1038/s41541-022-00580-z

**Published:** 2022-12-19

**Authors:** Juan Shi, Gang Wang, Jian Zheng, Abhishek K. Verma, Xiaoqing Guan, Moffat M. Malisheni, Qibin Geng, Fang Li, Stanley Perlman, Lanying Du

**Affiliations:** 1 Institute for Biomedical Sciences, Georgia State University, Atlanta, GA USA; 2 Department of Microbiology and Immunology, University of Iowa, Iowa City, IA USA; 3 Department of Pharmacology, University of Minnesota Medical School, Minneapolis, MN USA; 4 Center for Coronavirus Research, University of Minnesota, Minneapolis, MN USA; 5 Department of Pediatrics, University of Iowa, Iowa City, IA USA; 6 Present Address: Department of Microbiology and Immunology, Center for Predictive Medicine, University of Louisville, Louisville, KY USA

**Keywords:** SARS-CoV-2, Protein vaccines

## Abstract

The SARS-CoV-2 Omicron variant harbors more than 30 mutations in its spike (S) protein. Circulating Omicron subvariants, particularly BA5 and other variants of concern (VOCs), show increased resistance to COVID-19 vaccines that target the original S protein, calling for an urgent need for effective vaccines to prevent multiple SARS-CoV-2 VOCs. Here, we evaluated the neutralizing activity and protection conferred by a BA1-S subunit vaccine when combined with or used as booster doses after, administration of wild-type S protein (WT-S). A WT-S/BA1-S cocktail, or WT-S prime and BA1-S boost, induced significantly higher neutralizing antibodies against pseudotyped Omicron BA1, BA2, BA2.12.1, and BA5 subvariants, and similar or higher neutralizing antibodies against the original SARS-CoV-2, than the WT-S protein alone. The WT-S/BA1-S cocktail also elicited higher or significantly higher neutralizing antibodies than the WT-S-prime-BA1-S boost, WT-S alone, or BA1-S alone against pseudotyped SARS-CoV-2 Alpha, Beta, Gamma, and Delta VOCs, and SARS-CoV, a closely related beta-coronavirus using the same receptor as SARS-CoV-2 for viral entry. By contrast, WT-S or BA1-S alone failed to induce potent neutralizing antibodies against all these viruses. Similar to the WT-S-prime-BA1-S boost, the WT-S/BA1-S cocktail completely protected mice against the lethal challenge of a Delta variant with negligible weight loss. Thus, we have identified an effective vaccination strategy that elicits potent, broadly, and durable neutralizing antibodies against circulating SARS-CoV-2 Omicron subvariants, other VOCs, original SARS-CoV-2, and SARS-CoV. These results will provide useful guidance for developing efficacious vaccines that inhibit current and future SARS-CoV-2 variants to control the COVID-19 pandemic.

## Introduction

Severe acute respiratory coronavirus-2 (SARS-CoV-2), which causes Coronavirus Disease 2019 (COVID-19), has resulted in devastating damage to human health and to the global economy. The genome of SARS-CoV-2 encodes four structural proteins, including spike (S), membrane (M), envelope (E), and nucleocapsid (N) proteins, among which the S protein plays a critical role in viral infection and pathogenesis^1,2^; thus, it is a major vaccine and therapeutic target^2–4^. The S protein comprises S1 and S2 subdomains: the receptor-binding domain (RBD) within the S1 subdomain binds to a cellular receptor, angiotensin-converting enzyme 2 (ACE2), after which the S2 domain initiates fusion between the viral and cell membranes to mediate viral entry into host cells^2,5,6^. The S protein has a trimeric structure, with three RBD molecules in the up or down positions; only the RBD in the up position can bind the ACE2 receptor^7,8^.

**Fig. 1:**
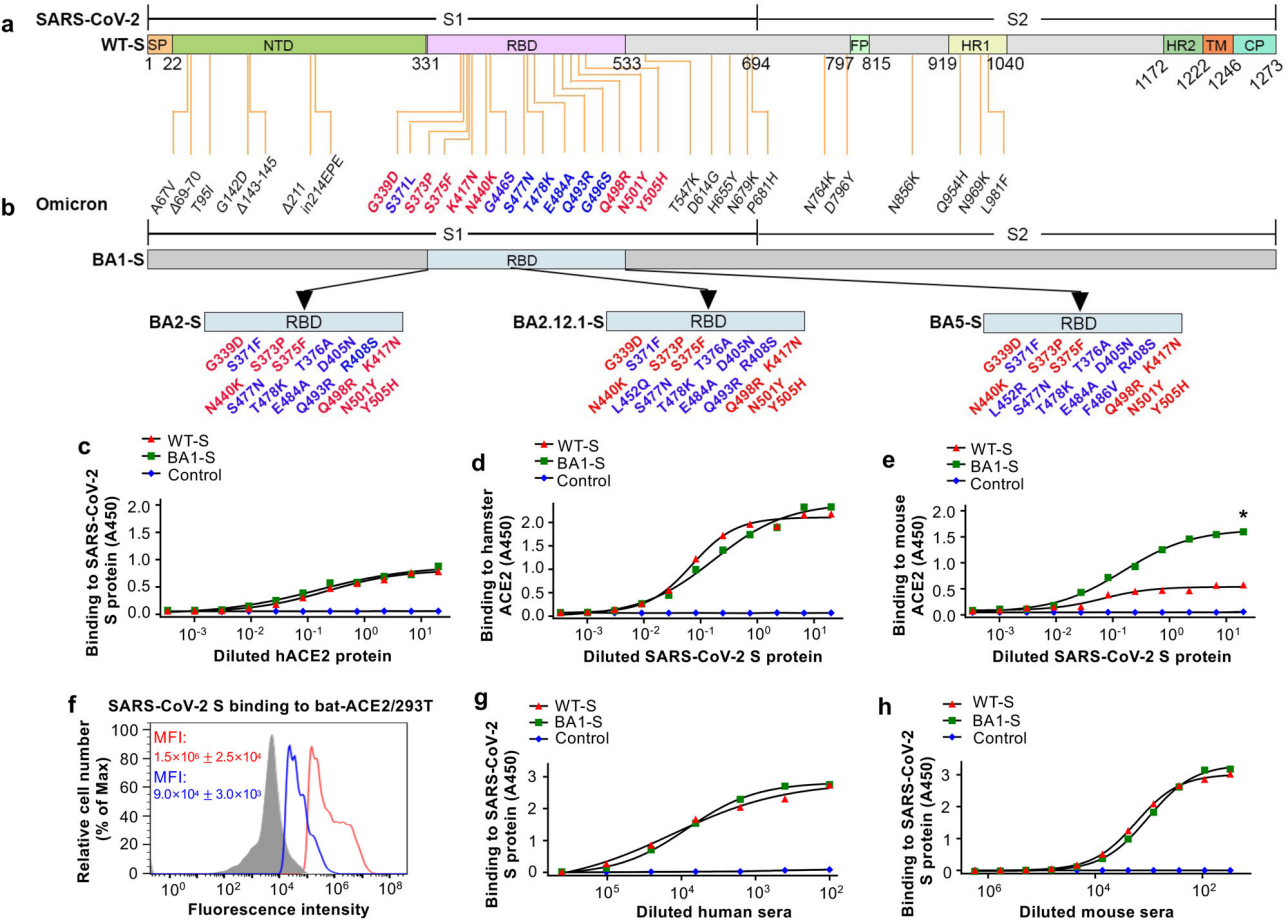
Schematic map of spike (S) protein of SARS-CoV-2 original strain and Omicron subvariants and characterization of SARS-CoV-2 BA1-S protein. Schematic map of SARS-CoV-2 S protein of the original wild-type (WT-S) strain (**a**) and Omicron BA1 subvariant (BA1-S) (**b**). Mutant amino acid residues of Omicron BA1 subvariant are shown in the S1 (including N-terminal domain (NTD) and receptor-binding domain (RBD)) and S2 subunits of S protein, respectively. Mutant amino acid residues in the RBD of Omicron BA2, BA2.12.1, and BA5 are shown (**b**). SP signal peptide. FP fusion peptide. HR1 and HR2, heptad repeat regions 1 and 2. TM transmembrane domain. CP cytoplasmic tail. ELISA analysis of binding of Omicron BA1-S protein (BA1-S) or original S protein (WT-S) to human angiotensin-converting enzyme 2 (hACE2) (**c**), hamster ACE2 (**d**), and mouse ACE2 (**e**) proteins, respectively. Control, PBS. Statistical significance between the binding of WT-S and BA1-S proteins to mouse ACE2 protein was analyzed using a two-tailed student *t*-test, and * (*P* < 0.05) indicates a significant difference. **f** Flow cytometry analysis of binding of BA1-S (blue line) and WT-S (red line) proteins to bat ACE2-expressing 293T cells (bat-ACE2/293T). 293T cells were transiently transfected with bat ACE2 plasmid and incubated with each protein (5 µg/ml) for analysis by flow cytometry. Gray shading, mock-incubated cells. MFI, median fluorescence intensity. ELISA for detection of binding of WT-S and BA1-S proteins to SARS-CoV-2 S-vaccinated human (**g**) and mouse (**h**) serum-neutralizing antibodies, respectively. The data (in **c**–**h**) are expressed as mean ± standard deviation of the mean (s.e.m.) of the duplicate to quadruple wells. The experiments were repeated twice, leading to similar results.

Since the emergence of SARS-CoV-2, a variety of variants of concern (VOCs) have been identified. These variants include previously circulating VOCs Alpha (B.1.1.7), Beta (B.1.351), Gamma (P.1), and Delta (B.1.617.2), and the recently circulating VOC Omicron (B.1.1.529) and its BA.1, BA.2 (BA2.12.1), BA.3, BA.4, and BA.5 subvariants^9^, which have high transmissibility due to many mutations in the S protein^10,11^. Compared with the original SARS-CoV-2 strain, Omicron BA1 has about 38 amino acid substitutions in the S protein, 15 of which are in the RBD region (Fig. 1a, b). Other Omicron subvariants, such as BA2, BA2.12.1, and BA5, also harbor many different amino acid variations in the RBD of the S protein (Fig. 1a, b).

Most of these SARS-CoV-2 variants, particularly the currently circulating Omicron subvariants, are resistant to neutralizing antibodies induced by the first-generation COVID-19 vaccines that target the S protein of the original SARS-CoV-2 strain^12–17^. Thus, there is an urgent need to develop new vaccines to prevent the further spread of COVID-19 caused by VOCs and contain the pandemic. SARS-CoV, a coronavirus belonging to the same beta-coronavirus genus as SARS-CoV-2, caused a global outbreak during 2002–2003; this virus also uses ACE2 as a cellular receptor for vial entry^18,19^. Here, we developed an effective vaccination strategy using a SARS-CoV-2 S protein cocktail that induces potent and durable neutralizing antibody responses against four Omicron subvariants (BA1, BA2, BA2.12.1, and BA5) and other SARS-CoV-2 VOCs (Alpha, Beta, Gamma, and Delta), in addition to the original SARS-CoV-2 and SARS-CoV viruses.

## Results

### Characterization of SARS-CoV-2 Omicron BA1-S protein

A recombinant BA1-S expressing the S protein of Omicron BA1, along with HexaPro sequences and a C-terminal foldon sequence (BA1-S), was constructed. In addition, a recombinant WT-S control was constructed to express the S protein of the original SARS-CoV-2 wild-type strain harboring the D614G mutation. Both proteins were expressed in HEK293F cells, purified from cell culture supernatants, and tested for binding to ACE2 receptors from different species. The results showed that the BA1-S protein bound to soluble human ACE2 (hACE2) and hamster ACE2 proteins with binding similar to, or slightly higher than, that of WT-S; however, it bound to mouse ACE2 protein with significantly higher affinity than WT-S (Fig. 1c–e). Nevertheless, the binding of BA1-S to cell-associated bat ACE2 was much lower than that of WT-S (Fig. 1f, Supplementary Fig. 1). Similar to WT-S, the BA1-S protein bound efficiently to SARS-CoV-2 vaccinated human and mouse serum neutralizing antibodies (Fig. 1g, h). These data suggest that BA1-S protein binds to the SARS-CoV-2 ACE2 receptor in humans, hamsters, and mice, and was antigenic (i.e., it was bound by antibodies in the serum of SARS-CoV-2-vaccinated humans and mice).

### Effective vaccination strategy induced potent and durable neutralizing antibodies against four Omicron subvariants and original SARS-CoV-2

**Fig. 2:**
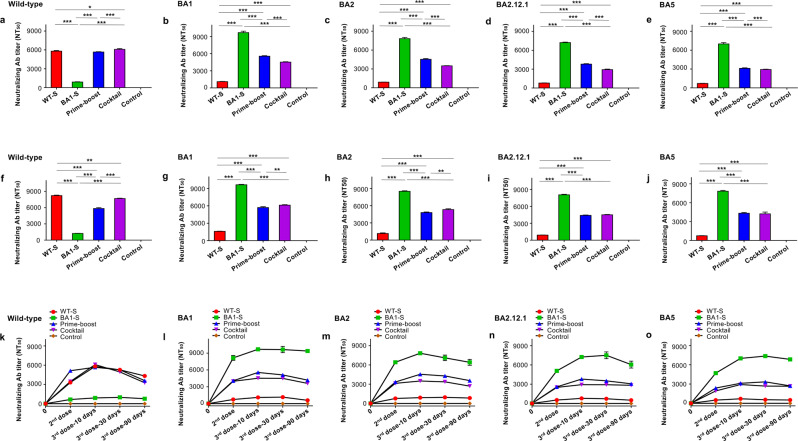
Cocktail or prime-boost of SARS-CoV-2 WT-S and BA1-S proteins induced potent and durable neutralizing antibodies against four Omicron subvariants and original SARS-CoV-2. BALB/c and K18-hACE2-Tg mice were immunized with WT-S, BA1-S, WT-S prime and BA1-S boost, WT-S and BA1-S cocktail, or PBS control in the presence of adjuvants 3 times at a 3-week interval. Sera were collected 10 days after the second immunization and 10, 30, and 90 days, respectively, after the third immunization for the detection of neutralizing antibodies against pseudotyped viruses encoding the S protein of the original SARS-CoV-2 strain and Omicron subvariants. NT_50_ was expressed as 50% neutralizing antibody titers against pseudovirus infection in 293T cells expressing the hACE2 receptor (hACE2/293T). NT_50_ against pseudotyped SARS-CoV-2 original wild-type strain (**a**, **f**), BA1 (**b**, **g**), BA2 (**c**, **h**), BA2.12.1 (**d**, **i**), and BA5 (**e**, **j**) 10 days after the third immunization in BALB/c (**a**–**e**) and K18-hACE2-Tg (**f**–**j**) mice, respectively. NT_50_ against the afore-mentioned pseudotyped SARS-CoV-2 original wild-type strain (**k**), BA1 (**l**), BA2 (**m**), BA2.12.1 (**n**), and BA5 (**o**) 10 days after the second immunization, as well as 10, 30, and 90 days, respectively, after the third immunization. The data are expressed as mean ± s.e.m. of quadruple wells from pooled sera of five mice in each group. Statistical significance among different vaccination groups was analyzed using Ordinary one-way ANOVA, and * (*P* < 0.05), ** (*P* < 0.01), and *** (*P* < 0.001) indicate significant differences. The experiments were repeated twice, leading to similar results.

To identify an effective vaccination strategy capable of inducing highly potent neutralizing antibodies against SARS-CoV-2 Omicron subvariants, we immunized BALB/c and K18-hACE2-transgenic (Tg) mice three times with the following proteins (10 μg/mouse) in the presence of adjuvants: (1) WT-S; (2) BA1-S; (3) one dose of WT-S, followed by two doses of BA1-S; and (4) a WT-S/BA1-S cocktail. Mice injected with phosphate-buffered saline (PBS) and adjuvants alone were included as a control. Sera were collected 10 days after the second and third doses, and then 30 and 90 days after the third dose, to evaluate neutralizing activity against pseudotyped viruses expressing the S protein of Omicron subvariants or the original virus. Pseudoviruses encoding the S proteins of BA2, BA2.12.1, and BA5 were constructed by mutation of amino acids in the RBD region (based on the BA1 S protein backbone) (Fig. 1a, b). The results from both mouse strains indicated that WT-S or BA1-S alone elicited high-titer neutralizing antibodies against the original SARS-CoV-2 (for WT-S) (Fig. 2a, f) or Omicron subvariants (for BA1-S) (Fig. 2b–e, g–j), but not against both the original SARS-CoV-2 and the Omicron subvariants. By contrast, the WT-S prime and BA1-S boost protocol, and the WT-S/BA1-S cocktail, induced significantly higher titers of neutralizing antibodies against the BA1, BA2, BA2.12.1, and BA5 subvariants than the WT-S protein (Fig. 2b–e, g–j), as well as similar or higher levels of neutralizing antibodies against the original SARS-CoV-2 strain (Fig. 2a, f). Of note, the WT-S/BA1-S cocktail induced higher or significantly higher neutralizing antibodies against the four Omicron subvariants (for K18-hACE2-Tg mice) (Fig. 2g–j) and the original SARS-CoV-2 (for BALB/c and K18-hACE2-Tg mice) (Fig. 2a, f) than the WT-S prime and BA1-S boost protocol. Compared with two doses, the third dose of these vaccines increased the titers of neutralizing antibodies against the original SARS-CoV-2 and all of the Omicron subvariants tested, and these neutralizing antibodies were maintained at high levels for at least 90 days after the third dose (Fig. 2k–o). Nevertheless, the PBS control induced background or undetectable levels of neutralizing antibodies against these pseudoviruses (Fig. 2). These data suggest that unlike the WT-S protein alone, the prime-boost protocol, particularly the WT-S/BA1-S cocktail, induced potent and durable neutralizing antibody titers against all four Omicron subvariants tested, as well as the original SARS-CoV-2 strain, whereas the BA1-S alone only elicited potent neutralizing antibodies against the Omicron subvariants, but not against the original SARS-CoV-2.

### Effective vaccination strategy induced broadly and durable neutralizing antibodies against other SARS-CoV-2 VOCs and SARS-CoV

**Fig. 3:**
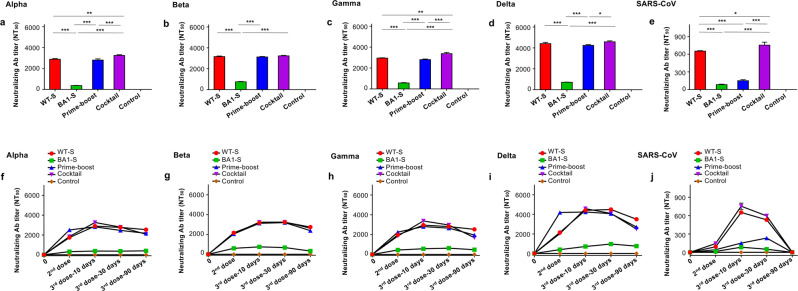
Cocktail or prime-boost of SARS-CoV-2 WT-S and BA1-S proteins induced potent and durable neutralizing antibodies against other SARS-CoV-2 VOCs and SARS-CoV. BALB/c mouse sera collected 10 days after the second immunization and, 10, 30, and 90 days, respectively, after the third immunization of WT-S, BA1-S, WT-S prime and BA1-S boost, WT-S and BA1-S cocktail, or PBS control were evaluated for neutralizing antibodies against pseudotyped viruses encoding S protein of SARS-CoV-2 Alpha variant containing 10 amino acid mutations (69–70 deletions, 145 deletion, N501Y, A570D, D614G, P681H, T716I, S982A, and D1118H) in the S protein, Beta/Gamma variants containing K417N/T, E484K, and N501Y mutations in the RBD, and Delta variant containing L452R, T478K, and P681R mutations in the S1 subunit, as well as the original SARS-CoV. NT_50_ was expressed as 50% neutralizing antibody titers against pseudovirus infection in hACE2/293T cells. NT_50_ against pseudotyped SARS-CoV-2 Alpha (**a**), Beta (**b**), Gamma (**c**), Delta (**d**) variants, and SARS-CoV (**e**) 10 days after the third immunization, as well as against pseudotyped SARS-CoV-2 Alpha (**f**), Beta (**g**), Gamma (**h**), Delta (**i**) variants, and SARS-CoV (**j**) 10 days after the second immunization and 10, 30, and 90 days, respectively, after the third immunization. The data are expressed as mean ± s.e.m of quadruple wells from pooled sera of five mice in each group. Statistical significance among different vaccination groups was analyzed using Ordinary one-way ANOVA, and * (*P* < 0.05), ** (*P* < 0.01), and *** (*P* < 0.001) indicate significant differences. The experiments were repeated twice, leading to similar results.

Sera from vaccinated mice were then tested for their ability to neutralize other SARS-CoV-2 VOCs and SARS-CoV. Pseudoviruses expressing the S protein of the SARS-CoV-2 Alpha, Beta, Gamma, and Delta variants, as well as that of SARS-CoV, were used for the neutralization tests. The results showed that the WT-S/BA1-S cocktail elicited higher or significantly higher neutralizing antibody titers than WT-S alone, or the WT-S prime and BA1-S boost protocol, against the SARS-CoV-2 Alpha, Beta, Gamma, and Delta variants, whereas the WT-S prime and BA1-S boost protocol induced neutralizing antibody titers similar to those of WT-S alone against these viruses (Fig. 3a–d). In addition, the WT-S/BA1-S cocktail elicited significantly higher antibody titers than WT-S alone, or the WT-S prime and BA1-S boost protocol, against pseudotyped SARS-CoV (Fig. 3e). The high-titer neutralizing antibodies against these SARS-CoV-2 VOCs have maintained for at least 90 days post-3rd immunization (Fig. 3f–i), and against SARS-CoV were maintained for more than 30 days after the third dose (Fig. 3j). Notably, BA1-S elicited the lowest titer of neutralizing antibodies against these viruses, significantly lower than that induced by the other vaccination groups (Fig. 3). By contrast, the PBS control elicited only background or undetectable levels of neutralizing antibodies against these viruses (Fig. 3). The above data suggest that, unlike the BA1-S protein, the prime-boost, particularly the WT-S/BA1-S cocktail, induce broad and durable neutralizing antibody responses against other SARS-CoV-2 VOCs and SARS-CoV.

### Effective vaccination strategy completely protected mice against Delta variant with significantly less weight loss

**Fig. 4:**
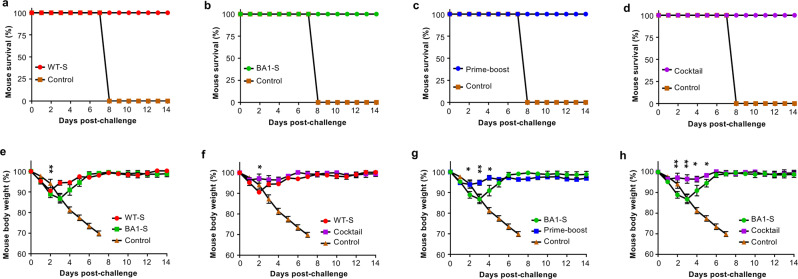
Cocktail or prime-boost of SARS-CoV-2 WT-S and BA1-S proteins completely protected mice from the lethal Delta variant challenge. Above immunized K18-hACE2-Tg mice or control mice injected with PBS plus adjuvants were challenged with the SARS-CoV-2 Delta variant (10,000 PFU/mouse) 30 days after the last immunization, and observed for survival (**a**–**d**) and body weight changes (**e**–**h**) for a period of 14 days after challenge. The data (in **e**–**h**) are expressed as mean ± s.e.m of five mice in each group. Statistical significance among different vaccination groups was analyzed using Ordinary one-way ANOVA. * (*P* < 0.05) and ** (*P* < 0.01) indicate significant differences between the WT-S and BA1-S (**e**), WT-S and WT-S/BA1-S cocktail (**f**), BA1-S and WT-S prime/BA1-S boost (**g**), as well as BA1-S and WT-S/BA1-S cocktail (**h**) groups.

Among SARS-CoV-2 variants, the Delta variant shows increased disease severity and mortality^20–22^. To investigate the protective efficacy of the WT-S/BA1-S or prime-boost vaccination strategy against Delta variant-caused death and weight loss, immunized K18-hACE2-Tg mice were challenged with a lethal dose of SARS-CoV-2 Delta variant 30 days after the last immunization, and survival and weight changes were monitored for 14 days. Mice in the PBS control group showed continuous weight loss, and all died by Day 8 post-virus challenge (Fig. 4a–h), whereas all mice in the vaccination groups survived after the challenge with the Delta variant (Fig. 4a–d). In contrast to significant weight loss (especially on Days 2–5 after challenge) in mice immunized with WT-S or BA1-S alone, the weights of mice immunized using the WT-S/BA1-S cocktail or prime-boost regimens remained unchanged (Fig. 4e–h). These data suggest that similar to the WT-S and BA1-S prime-boost, the WT-S/BA1-S cocktail provided the best protection against weight loss after the challenge with the Delta variant.

## Discussion

SARS-CoV-2 Omicron subvariant BA5 is among the recent dominant VOCs circulating in the United States and other countries^23,24^. Although the severity of BA4 and BA5 is similar to that of other Omicron subvariants such as BA1 and BA2, they are more transmissible as current vaccines are less effective against them; increased infections lead inevitably to increased hospitalizations and deaths^24^. The Delta variant also showed higher transmissibility, disease severity, mortality, and hospitalizations than other VOCs such as Alpha and Beta^21,25^. Most of these VOCs demonstrate resistance to currently available COVID-19 vaccines^12,26,27^; this is because they escape immunity induced by these vaccines as they were designed to target the original SARS-CoV-2 strain. Therefore, there is an urgent need to develop effective vaccines capable of inducing balanced immune responses against these VOCs, particularly dominant Omicron BA5, in addition to the original virus strain.

Here, we generated a subunit vaccine based on the S protein of the Omicron BA1 subvariant and evaluated its neutralizing activity; it was evaluated on its own, in combination with the original S protein, and as a booster after vaccination with the original S protein. The Omicron BA1-S protein alone induced high titers of neutralizing antibodies against Omicron subvariants BA1, BA2, BA2.12.1, and BA5, but not against other VOCs or the original SARS-CoV-2. However, the original S protein alone elicited potent neutralizing antibodies against the original SARS-CoV-2, and lower titers against the Alpha, Beta, Gamma, and Delta variants, but it did not induce effective antibodies against Omicron subvariants. By contrast, a WT-S prime and BA1-S boost protocol, particularly the WT-S/BA1-S cocktail, elicited high titers of neutralizing antibodies against all VOCs tested (the four Omicron subvariants, and the Alpha, Beta, Gamma, and Delta variants), as well as the original SARS-CoV-2. In particular, the WT-S/BA1-S cocktail induced more durable and higher titers of antibodies than the WT-S prime and BA1-S boost protocol against the Alpha, Beta, Gamma, and Delta SARS-CoV-2 VOCs, as well as against SARS-CoV.

Of note, the overall titers of neutralizing antibodies elicited by the WT-S prime and BA1-S boost, or WT-S/BA1-S cocktail, against SARS-CoV-2 Omicron subvariants, including the BA5 subvariant, were lower than those against the original SARS-CoV-2 strain. These data are consistent with the reported vaccines, such as those based on mRNAs, viral vectors, and inactivated viruses, which showed reduced neutralizing activity against the Omicron or its subvariants as compared to the original virus isolate or other VOCs^28–31^. Similarly, skin-patch delivery of a HexaPro S protein of the original SARS-CoV-2 strain elicited higher neutralizing antibody titers against the Gamma and Delta VOCs and original strain, but relatively lower titers of neutralizing antibodies against the Omicron VOC^32^. Previously reported vaccines demonstrated folded reduction of neutralizing antibodies against the Omicron BA4 and BA5 subvariants than against the BA1, BA2, or BA2.12.1 subvariant^28,31,33^. In comparison, we found that the titers of neutralizing antibodies induced by the WT-S prime and BA1-S boost, or WT-S/BA1-S cocktail, against currently circulating BA5 were not significantly lower than those against the BA1, BA2, and BA2.12.1 subvariants, which were maintained for several months after the last vaccination, suggesting their potent ability to prevent infection of these Omicron subvariants. Notably, the Omicron BA4 subvariant encodes the same amino acid mutations as BA5 in the S protein RBD region, indicating that vaccine-induced antibodies will also effectively neutralize the BA4 subvariant.

This study also evaluated the in vivo protective efficacy of the above vaccination strategies against infection of the Delta variant due to its increased mortality than other VOCs or the original strain^20,22,34^. In either WT-S prime and BA1-S boost or WT-S/BA1-S cocktail protocol, the induced neutralizing antibodies were sufficient to completely protect immunized mice against lethal challenge with the Delta variant, without obvious weight loss. One limitation of this report is that it did not evaluate the protection of these vaccination strategies in reducing viral loads or histopathological effects after a challenge with the Delta or current circulating variant strains. Such comparisons will be warranted in future studies, as these observations will provide important information for evaluating the vaccine’s ability to prevent viral replication and pathogenesis, in consideration of all vaccinated mice were protective against virus-caused mortality.

In conclusion, we developed an effective vaccination strategy to elicit balanced neutralizing antibody responses against currently circulating Omicron subvariants, including BA5, and other SARS-CoV-2 variants. The results serve as useful guidance for the development of efficacious vaccines that will prevent infection by current and future SARS-CoV-2 variants, and control the COVID-19 pandemic.

## Methods

### Construction, expression, and purification of recombinant proteins

The DNA-S sequence of SARS-CoV-2 Omicron (B.1.1.529, BA1) variant (BA1-S) was amplified by polymerase chain reaction (PCR) using a codon-optimized plasmid encoding S protein with HexaPro sequences of SARS-CoV-2 Omicron (GISAID accession number EPI_ISL_6795835). The DNA-S sequence of SARS-CoV-2 wild-type (WT-S) was amplified by PCR using a plasmid encoding codon-optimized S protein of the original SARS-CoV-2 strain (GenBank accession number QHR63250.2). The amplified PCR fragments containing a C-terminal foldon trimerization domain and His_6_ tag were inserted into pLenti expression vector. D614G mutation was further added to the WT-S plasmid. The recombinant plasmids were transfected into HEK293F cells, and the related proteins were purified from the culture supernatants using Ni-NTA Superflow (Qiagen).

### Enzyme-linked immunoassay (ELISA)

ELISA was used to measure the binding between each S protein and ACE2 proteins from different species^35,36^. For binding to human ACE2 (hACE2) protein, 96-well ELISA plates were coated with purified BA1-S or WT-S protein (1 μg/ml) at 4 °C overnight, and blocked with 2% non-fat milk in PBST (PBS containing 0.05% Tween-20) for 1 h at 37 °C. The plates were then incubated with serial dilutions of hACE2 protein (Laboratory stock) for 1 h at 37 °C. After three washes with PBST, the plates were sequentially incubated with goat anti-hACE2 IgG antibody (0.2 μg/ml, R&D System AF933) and horseradish peroxidase (HRP)-conjugated rabbit anti-goat IgG antibody (1:5000 dilution, Abcam ab6741). For binding to mouse ACE2 and hamster ACE2, ELISA plates were coated with respective mouse ACE2 (1 μg/ml, R&D System 3437-ZN) or hamster ACE2 (1 μg/ml, R&D System 10578-ZN) protein, and then sequentially incubated with serial dilutions of each S protein, SARS-CoV-2 S-specific mouse polyclonal antibody (1:2,000 dilution, Laboratory stock), and anti-mouse IgG-Fab-HRP antibody (1:5000 dilution, Sigma A9917-1ML) for 1 h at 37 °C. After further washes, the respective plates were incubated with substrate TMB (3,3′,5,5′-Tetramethylbenzidine) (Sigma), and 1 N H_2_SO_4_ was added to stop the reaction. The A450 value (absorbance at 450 nm) was measured by Cytation 7 Microplate Multi-Mode Reader and Gen5 software (BioTek Instruments).

ELISA was also used to detect the binding between BA1-S or WT-S protein and SARS-CoV-2 vaccinated human or mouse sera^36,37^. Specifically, ELISA plates were coated with each purified S protein (1 μg/ml), and then sequentially incubated with serial dilutions of human sera from SARS-CoV-2 S-mRNA vaccination or mouse sera from SARS-CoV-2 S-trimer protein immunization. After washes, the plates were incubated with anti-human-IgG-Fab-HRP (1:5000 dilution, Abcam ab87422) or anti-mouse IgG-Fab-HRP (1:5000 dilution, Sigma A9917-1ML) antibody for 1 h at 37 °C. Other procedures were carried out as described above.

### Flow cytometry

Flow cytometry analysis was carried out to detect the binding between BA1-S or WT-S protein and bat ACE2 receptor in bat ACE2-expressing 293T cells as described below^35^. Specifically, 293T cells were transiently transfected with bat ACE2 plasmid using the polyetherimide (PEI) method. Forty-eight hours later, the transfected cells were incubated with each purified protein (5 µg/ml) for 30 min at room temperature and stained with FITC-conjugated anti-His IgG antibody (1:10 dilution, Invitrogen MA1-81891). After washes and fixation with Fixation/Permeabilization Concentrate reagent (Invitrogen), the cells were analyzed by CytoFLEX flow cytometer (Beckman Coulter Life Sciences), and the data were processed with FlowJo (V10.0).

### Construction of recombinant plasmids for a package of pseudovirus

Recombinant plasmids respectively encoding S protein of the original SARS-CoV-2 strain (GenBank accession number QHR63250.2) and Alpha (B.1.1.7) variant (GISAID accession number EPI_ISL_718813), as well as the original SARS-CoV strain (GenBank accession number AY274119), were constructed by inserting respective DNA sequences into pcDNA3.1/V5-His-TOPO vector (Thermo Fisher Scientific)^38^. Omicron (B.1.1.529) variants BA1 (GISAID accession number EPI_ISL_6795835), BA2 (GISAID accession number EPI_ISL_12030355), BA2.12.1 (GISAID accession number EPI_ISL_12061569), and BA5 (GISAID accession number EPI_ISL_12043290), as well as other recombinant plasmids expressing S protein of Beta (B.1.351), Gamma (P.1), and Delta (B.1.617.2) variants, which contain single or multiple amino acid substitutions at the RBD, were constructed using multi-site-directed mutagenesis kit (Agilent Technologies). The recombinant plasmids were confirmed for correct sequences and used for the generation of pseudoviruses.

### Pseudovirus generation and neutralization assay

Pseudoviruses were generated as described below^36,38–40^. Specifically, the plasmid encoding original or mutant S protein was co-transfected with pLenti-CMV-luciferase and PS-PAX2 plasmids (Addgene) into 293T cells using the PEI transfection method described above. Supernatants containing pseudoviruses were collected at 72 h after transfection and processed for the below pseudovirus neutralization assay. Pseudoviruses were incubated with serial dilutions of mouse sera for 1 h at 37 °C, and the virus-serum mixture was added to 96-well plates pre-seeded with 293T cells expressing hACE2 (hACE2/293T). Fresh medium was added to the cells 24 h later, which were sequentially incubated with cell lysis buffer, and luciferase substrate (Promega) 72 h later. Relative luciferase activity was measured using Cytation 7 Microplate Multi-Mode Reader and Gen5 software (BioTek Instruments). Pseudovirus neutralization was detected, based on which 50% neutralizing antibody titer (NT_50_) was calculated.

### Ethics statement

Female BALB/c mice (4-month-old) mice and K18-hACE2-transgenic (Tg) mice (6–8-week-old) were used in the study. The animal protocols were approved by the Institutional Animal Care and Use Committees (IACUC) of Georgia State University and the University of Iowa. All mouse-related experiments were carried out in strict accordance with the Guidelines for the Care and Use of Laboratory Animals of the National Institutes of Health and our approved protocols.

### Mouse immunization and sample collection

Mouse immunization was carried out as described below^36,41,42^. Specifically, mice were randomly assigned to each group, and intramuscularly (I.M., 100 μl/mouse) immunized with the following proteins or PBS: (1) BA1-S protein, (2) WT-S protein, (3) BA1-S (1st dose) and WT-S (2nd and 3rd doses), (4) BA1-S + WT-S cocktail (10 μg/mouse), or (5) PBS control, in the presence of aluminum (500 μg/mouse) and monophosphoryl lipid A (MPL, 10 μg/mouse) adjuvants (InvivoGen). The immunized mice were boosted twice at a 3-week interval, and sera were collected 10 days after the second and third immunizations and 30 and 90 days after the third immunization, respectively, for the detection of neutralizing antibodies, as described above.

### Challenge of mice with SARS-CoV-2 Delta variant

Thirty days after the last immunization (as described above), K18-hACE2-Tg mice were intranasally (I.N.) challenged with SARS-CoV-2 Delta variant (10,000 PFU/mouse, 50 μl/mouse), and observed for survival and body weight changes for up to 14 days after the challenge^36,38,43^. Mice with 25% weight loss and significant clinical symptoms, or 30% weight loss, were humanely euthanized by cervical dislocation under anesthesia.

### Statistical analysis

Statistical significances among different groups were calculated using GraphPad Prism 9 statistical software. Statistical significance between the binding of each S protein to mouse ACE2 receptor was calculated using a two-tailed student *t*-test. Neutralizing antibody titers and weight changes among different groups were calculated using Ordinary one-way ANOVA. *P* < 0.05 was considered as significant. *, **, and *** represent *P* < 0.05, *P* < 0.01, or *P* < 0.001.

### Reporting summary

Further information on research design is available in the Nature Research Reporting Summary linked to this article.

## Supplementary information

Supplementary Fig. 1

REPORTING SUMMARY

## Data Availability

All relevant data provided in this paper are available from the corresponding authors upon request. No special code was reported in this paper.
