# Supplementary material for: Effective vaccination strategy using SARS-CoV-2 spike cocktail against Omicron and other variants of concern
Source: NPJ Vaccines. 2022 Dec 19;7:169. doi: 10.1038/s41541-022-00580-z (PMC9762654; doi:10.1038/s41541-022-00580-z)
Supplement: Supplementary file 1 — Supplementary Fig. 1 [file 41541_2022_580_MOESM1_ESM.pdf]

Supplementary Figures

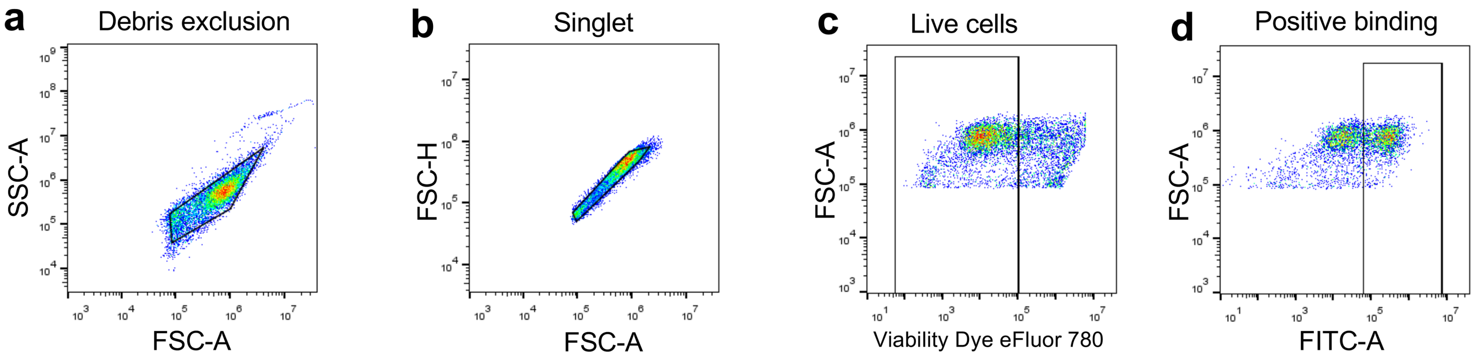

Supplementary Figure 1. Gating strategy to determine the binding of each S protein to bat ACE2-expressing 293T cells. (**a**) Forward and side scatter density plots for excluding debris. (**b**) Forward scatter height (FSC-H) versus forward scatter area (FSC-A) plot for doublet exclusion. (**c**) Fixable Viability Dye eFluor 780 was used to exclude dead cells. (**d**) FITC-A was used to identify cells that specifically bound to the target proteins.
