# Supplementary material for: Effective vaccination strategy using SARS-CoV-2 spike cocktail against Omicron and other variants of concern
Source: NPJ Vaccines. 2022 Dec 19;7:169. doi: 10.1038/s41541-022-00580-z (PMC9762654; doi:10.1038/s41541-022-00580-z)
Supplement: Supplementary file 2 — REPORTING SUMMARY [file 41541_2022_580_MOESM2_ESM.pdf]

nature portfolio

Corresponding author(s): Perlman S; Du, L

Last updated by author(s): Oct 15, 2022

Reporting Summary

Nature Portfolio wishes to improve the reproducibility of the work that we publish. This form provides structure for consistency and transparency in reporting. For further information on Nature Portfolio policies, see our Editorial Policies and the Editorial Policy Checklist.

Statistics

For all statistical analyses, confirm that the following items are present in the figure legend, table legend, main text, or Methods section.

n/a	Confirmed	
☐	☒	The exact sample size (*n*) for each experimental group/condition, given as a discrete number and unit of measurement
☐	☒	A statement on whether measurements were taken from distinct samples or whether the same sample was measured repeatedly
☐	☒	The statistical test(s) used AND whether they are one- or two-sided<br>*Only common tests should be described solely by name; describe more complex techniques in the Methods section.*
☒	☐	A description of all covariates tested
☒	☐	A description of any assumptions or corrections, such as tests of normality and adjustment for multiple comparisons
☐	☒	A full description of the statistical parameters including central tendency (e.g. means) or other basic estimates (e.g. regression coefficient) AND variation (e.g. standard deviation) or associated estimates of uncertainty (e.g. confidence intervals)
☒	☐	For null hypothesis testing, the test statistic (e.g. *F*, *t*, *r*) with confidence intervals, effect sizes, degrees of freedom and *P* value noted<br>*Give P values as exact values whenever suitable.*
☒	☐	For Bayesian analysis, information on the choice of priors and Markov chain Monte Carlo settings
☒	☐	For hierarchical and complex designs, identification of the appropriate level for tests and full reporting of outcomes
☒	☐	Estimates of effect sizes (e.g. Cohen's *d*, Pearson's *r*), indicating how they were calculated

Our web collection on statistics for biologists contains articles on many of the points above.

Software and code

Policy information about availability of computer code

Data collection	FlowJo (V10.0) from CytoFLEX flow cytometer was used to measure fluorescence intensity in flow cytometry analysis. Gen5 was used to measure luciferase activity in the pseudovirus neutralization assay and A450 value in ELISA.
Data analysis	FlowJo (V10.0) software, Gen5 software, and GraphPad Prism 9 statistical analysis software

For manuscripts utilizing custom algorithms or software that are central to the research but not yet described in published literature, software must be made available to editors and reviewers. We strongly encourage code deposition in a community repository (e.g. GitHub). See the Nature Portfolio guidelines for submitting code & software for further information.

Data

Policy information about availability of data

All manuscripts must include a data availability statement. This statement should provide the following information, where applicable:

- Accession codes, unique identifiers, or web links for publicly available datasets
- A description of any restrictions on data availability
- For clinical datasets or third party data, please ensure that the statement adheres to our policy

All data generated or analyzed during this study are included in the manuscript.

Human research participants

Policy information about studies involving human research participants and Sex and Gender in Research.

Reporting on sex and gender	N/A
Population characteristics	N/A
Recruitment	N/A
Ethics oversight	N/A

Note that full information on the approval of the study protocol must also be provided in the manuscript.

Field-specific reporting

Please select the one below that is the best fit for your research. If you are not sure, read the appropriate sections before making your selection.

☒ Life sciences ☐ Behavioural & social sciences ☐ Ecological, evolutionary & environmental sciences

For a reference copy of the document with all sections, see nature.com/documents/nr-reporting-summary-flat.pdf

Life sciences study design

All studies must disclose on these points even when the disclosure is negative.

Sample size	Five mice were used in each group. Such sample size was determined based on the prior studies indicating statistical significance among different groups.
Data exclusions	No data were excluded from the data analysis.
Replication	ELISA, neutralization assays, and other in vitro assays were repeated at least twice, leading to similar results.
Randomization	Mice were randomly assigned to each group for immunization.
Blinding	N/A

Reporting for specific materials, systems and methods

We require information from authors about some types of materials, experimental systems and methods used in many studies. Here, indicate whether each material, system or method listed is relevant to your study. If you are not sure if a list item applies to your research, read the appropriate section before selecting a response.

Materials & experimental systems

n/a	Involved in the study	
☐	☒	Antibodies
☐	☒	Eukaryotic cell lines
☒	☐	Palaeontology and archaeology
☐	☒	Animals and other organisms
☒	☐	Clinical data
☒	☐	Dual use research of concern

Methods

n/a	Involved in the study	
☒	☐	ChIP-seq
☐	☒	Flow cytometry
☒	☐	MRI-based neuroimaging

Antibodies

Antibodies used	Goat anti-hACE2 IgG antibody (R&D System), HRP-conjugated rabbit anti-goat IgG antibody (Abcam), anti-mouse IgG-Fab-HRP antibody (Sigma), anti-human-IgG-Fab-HRP (Abcam), and FITC-conjugated anti-His IgG antibody (Invitrogen)
Validation	These antibodies were validated in the prior studies for ELISA and flow cytometry analyses.

Eukaryotic cell lines

Policy information about cell lines and Sex and Gender in Research

Cell line source(s)	HEK293T cells (CRL-3216, ATCC); HEK293F cells (K900001, ThermoFisher Scientific); hACE2/293T cells (lab stock).
Authentication	Cells were confirmed for viability and morphology before use.
Mycoplasma contamination	No mycoplasma contamination was identified.
Commonly misidentified lines (See ICLAC register)	None.

Animals and other research organisms

Policy information about studies involving animals; ARRIVE guidelines recommended for reporting animal research, and Sex and Gender in Research

Laboratory animals	BALB/c mice (4-month-old) and K18-hACE2-transgenic mice (6-8-week-old) were used in the study.
Wild animals	N/A
Reporting on sex	Female mice were used. Prior studies did not show significant difference between the data from male and female mice.
Field-collected samples	N/A
Ethics oversight	The animal protocols were approved by our Institutional Animal Care and Use Committees (IACUC). All mouse-related experiments were carried out in strict accordance with the Guidelines for the Care and Use of Laboratory Animals of National Institutes of Health and our approved protocols.

Note that full information on the approval of the study protocol must also be provided in the manuscript.

Flow Cytometry

Plots

Confirm that:

- [x] The axis labels state the marker and fluorochrome used (e.g. CD4-FITC).
- [x] The axis scales are clearly visible. Include numbers along axes only for bottom left plot of group (a 'group' is an analysis of identical markers).
- [ ] All plots are contour plots with outliers or pseudocolor plots.
- [x] A numerical value for number of cells or percentage (with statistics) is provided.

Methodology

Sample preparation	HEK293T cells transiently transfected with bat-ACE2 plasmid were incubated with the target proteins, and then stained with FITC-conjugated anti-His-IgG antibody, followed by analysis by flow cytometry.
Instrument	CytoFLEX flow cytometer
Software	FlowJo (V10.0)
Cell population abundance	Single cells were selected by plotting FSC-H vs FSC-A. The dead cells were excluded using the viability dye, Fixable Viability Dye eFluor 780.
Gating strategy	Cells stained with anti-His-FITC antibody were used to determine the level of non-specific binding, which indicated the boundaries between positive and negative staining.

- [x] Tick this box to confirm that a figure exemplifying the gating strategy is provided in the Supplementary Information.
